# Emerging perspectives of copper-mediated transcriptional regulation in mammalian cell development

**DOI:** 10.1093/mtomcs/mfae046

**Published:** 2024-10-07

**Authors:** Fa'alataitaua M Fitisemanu, Teresita Padilla-Benavides

**Affiliations:** De partment of Molecular Biology and Biochemistry, Wesleyan University, CT 06459, United States; De partment of Molecular Biology and Biochemistry, Wesleyan University, CT 06459, United States

**Keywords:** transcriptional regulation, copper, metal homeostasis, copper-binding proteins, development, gene expression

## Abstract

Copper (Cu) is a vital micronutrient necessary for proper development and function of mammalian cells and tissues. Cu mediates the function of redox active enzymes that facilitate metabolic processes and signaling pathways. Cu levels are tightly regulated by a network of Cu-binding transporters, chaperones, and small molecule ligands. Extensive research has focused on the mammalian Cu homeostasis (cuprostasis) network and pathologies, which result from mutations and perturbations. There are roles for Cu-binding proteins as transcription factors (Cu-TFs) and regulators that mediate metal homeostasis through the activation or repression of genes associated with Cu handling. Emerging evidence suggests that Cu and some Cu-TFs may be involved in the regulation of targets related to development—expanding the biological roles of Cu-binding proteins. Cu and Cu-TFs are implicated in embryonic and tissue-specific development alongside the mediation of the cellular response to oxidative stress and hypoxia. Cu-TFs are also involved in the regulation of targets implicated in neurological disorders, providing new biomarkers and therapeutic targets for diseases such as Parkinson's disease, prion disease, and Friedreich's ataxia. This review provides a critical analysis of the current understanding of the role of Cu and cuproproteins in transcriptional regulation.

## Graphical Abstract

**Graphical Abstract:**
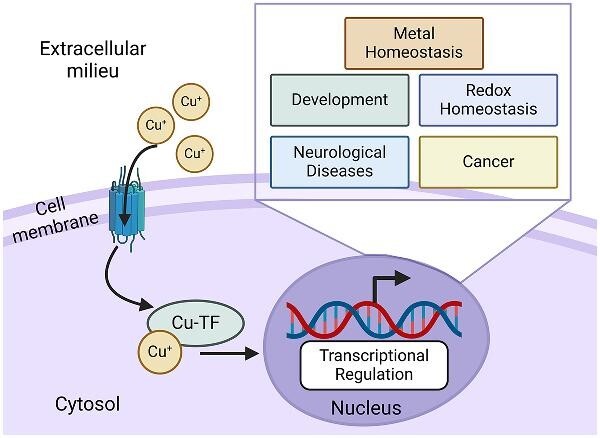
Emerging roles for copper and copper-binding transcription factors in mammalian cell development.

## Introduction

**Figure 1.**
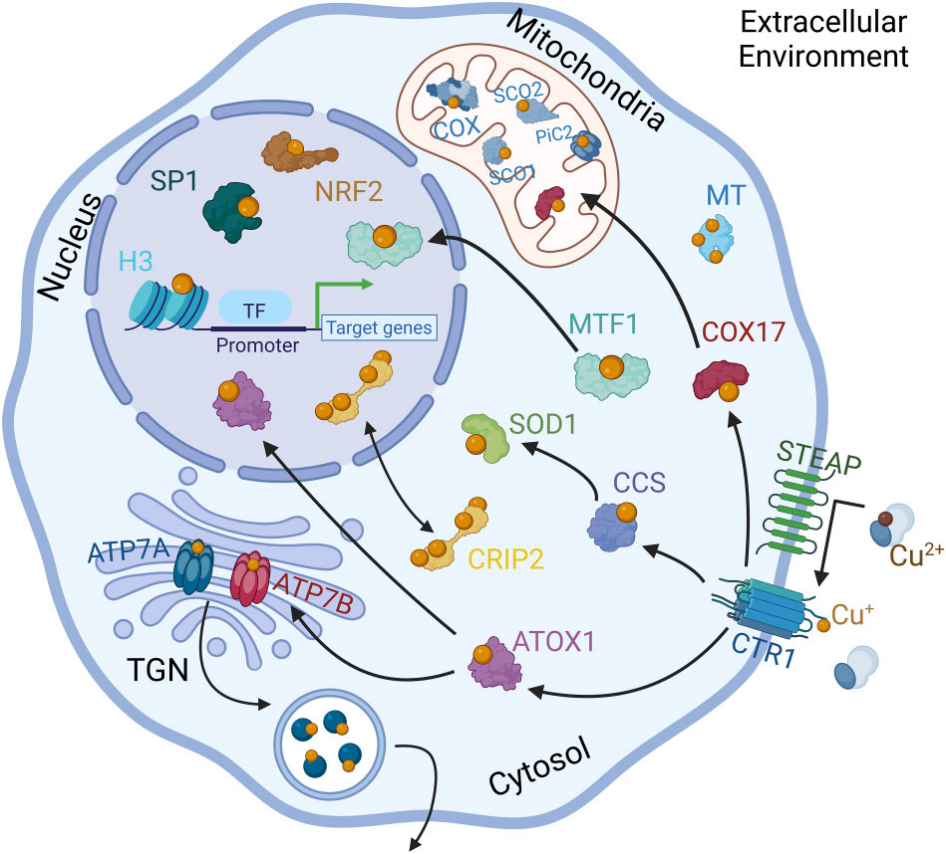
Schematic representation of Cu transporters, chaperones, and transcriptional regulators. Extracellular Cu^2+^ is reduced to a Cu^+1^ oxidation state and then imported by Cu-transporter CTR1 into the cytosol. Cu^+^ is delivered to cytosolic Cu chaperones, which mediate delivery to organelles such as the TGN, mitochondria, and nucleus. Cu is necessary for cellular respiration and redox activity in the mitochondria. The Cu^+^ P-type ATPases ATP7A and ATP7B mediate the export of Cu through the metalation of excreted cuproenzymes in the TGN. Cu-responsive transcription factors in the nucleus mediate Cu homeostasis and Cu-dependent development. Figure made with BioRender.

**Figure 2.**
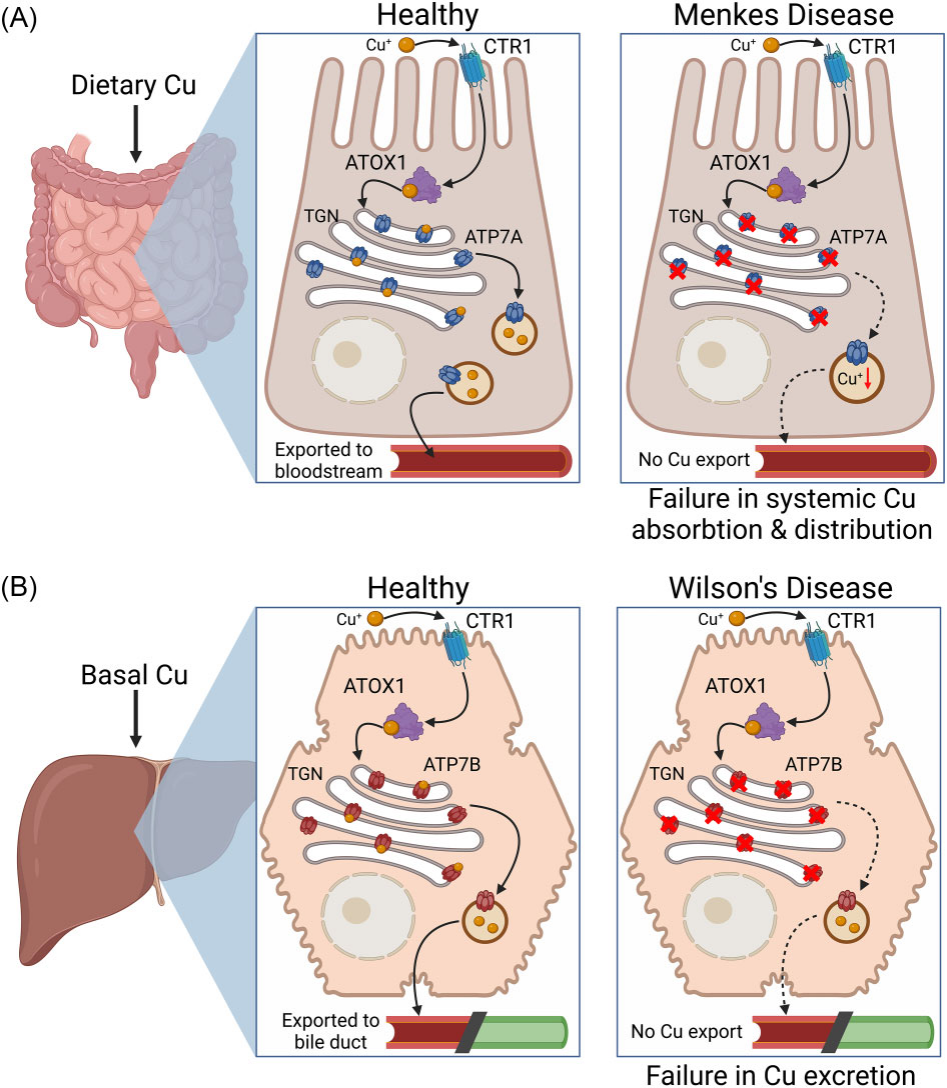
Schematic representation of Cu transport mediated by ATP7A and ATP7B. **A.** Left panel: Diagram of Cu transport through an enterocyte cell. CTR1 imports Cu through the apical membrane, from which Cu is trafficked by ATOX1 to the TGN. ATP7A mobilizes Cu into vesicles to metalate cuproproteins to be secreted into the bloodstream and extracellular milieu. Right panel: Menkes disease is characterized by mutations in ATP7A, which impairs absorption of dietary Cu, by impeding translocation from the cytosol into the bloodstream. This, in turn, causes systemic Cu deficiency. **B.** Left panel: Schematic representation of Cu transport through a hepatocyte cell. CTR1 transports Cu from the plasma through the basolateral membrane to ATOX1. ATOX1 chaperones Cu to the TGN where ATP7B facilitates metalation of excreted into the biliary tract. Right panel: Wilson's disease is caused by mutations in ATP7B, which abolishes biliary excretion of Cu, resulting in systemic accumulation of Cu that disrupts healthy cellular function. Figure made with BioRender.

Copper (Cu) is a trace element required for the proliferation, development, and function of mammalian cells and tissues [1]. Excessive cellular Cu levels result in the production of reactive oxygen species (ROS), which might disrupt protein, lipid, DNA, and iron–sulfur (Fe–S) cluster function [2]. Due to the reactivity of Cu, a broad network of transporters, chaperones, and transcriptional regulators maintains Cu homeostasis (cuprostasis, Fig. 1) [3, 4]. Failure in the cuprostatic network results in a variety of pathologies—including Menkes disease (MD), occipital horn syndrome, and Wilson's disease (WD), caused by mutations in the Cu^+^-P-type ATPases ATP7A and ATP7B (Fig. 2). Mutations in the *ATP7A* gene cause MD, resulting in neurological problems, convulsions, and abnormal development of bone, muscle, and connective tissue [5–7]. Occipital horn syndrome is a milder variant of MD, characterized by prominent connective tissue disorder [5, 6, 8, 9]. WD is caused by mutations in the *ATP7B* gene, with hallmarks including neurological symptoms, muscle stiffness, and liver disease [10–12]. Additional conditions associated with Cu disbalance include mitochondrial myopathies, and in recent years, Cu has also been associated with neuropathologies such as Parkinson's disease, prion diseases, and Friedreich's ataxia (FRDA) [13–16].

Cu-dependent mechanisms of cell proliferation and apoptosis—referred to as cuproplasia and cuproptosis, respectively—have recently been recognized as unique processes in cell growth and death under pathogenic conditions [17–24]. Cuproplasia is characterized by Cu-dependent cell proliferation and is associated with various cellular functions, spanning from mitochondrial respiration and antioxidant defense to redox signaling, kinase signaling, autophagy, and protein quality control [17]. Cu is enriched in cancer cells due to the energetic requirement for rapid proliferation and migration of cancerous cells, and Cu chelation has been used to treat cancer through mitigating cuproplasia [17, 19]. On the other hand, cuproptosis is distinct from other cell death mechanisms and occurs as a toxic gain of function in lipoylated mitochondrial tricarboxylic acid cycle (TCA) cycle proteins simulated by direct Cu binding [22]. This process results in the aggregation of lipoylated proteins. This aggregation disrupts Fe–S cluster proteins, leading to proteotoxic stress and cell death—in a similar manner to cell death caused by Cu toxicity in WD [20, 22]. The molecular mechanisms of cuproptosis have become a large focus of cancer and other disease research to develop novel diagnostics, prognostics, and treatments [25–31].

Research regarding Cu has been centered on metal homeostasis and the characterization of the biological impact of impairment of the cellular Cu network. Recent work, however, identified Cu as a necessary factor in the development and function of a variety of cell and tissue types, including brain, skeletal muscle, liver, and kidney [32–35]. These emerging studies point to a shift in focus toward non-canonical functions of Cu and cuproenzymes in the transcriptional regulation of cell and tissue development. This review outlines current research on the regulatory role of Cu and cuproproteins in transcription and development.

## Subcellular management of copper

**Figure 3.**
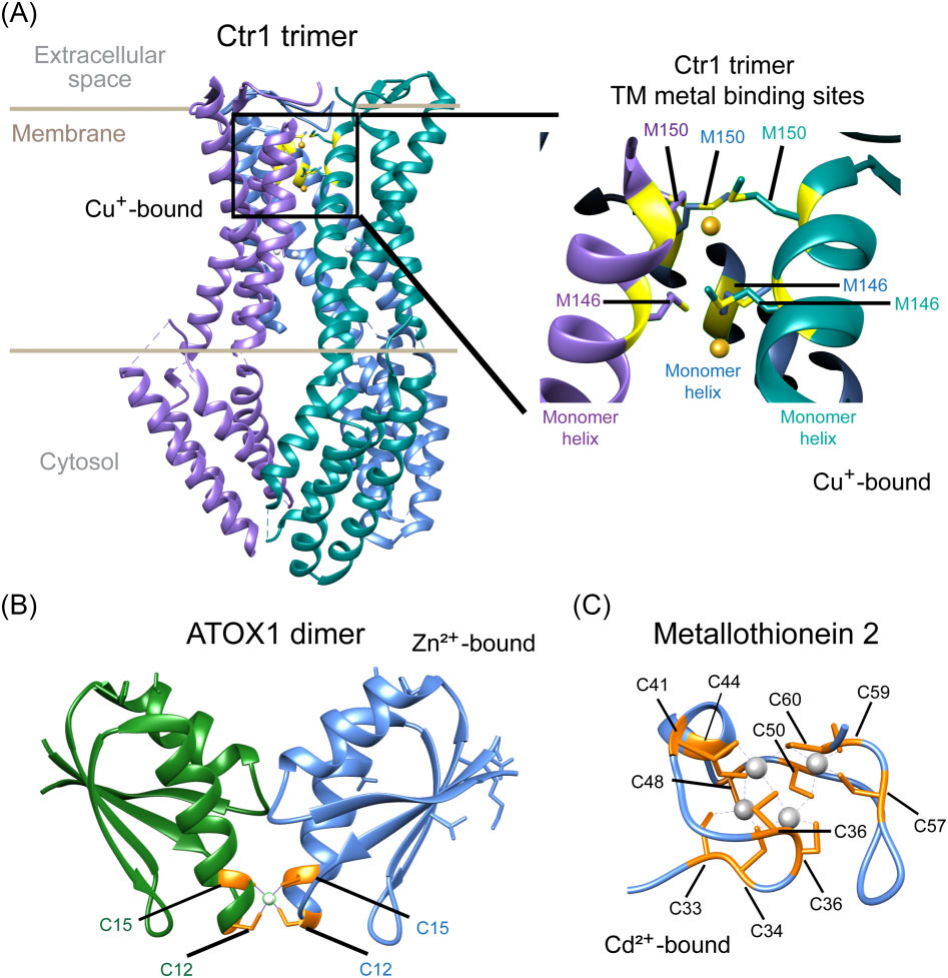
Protein structures of key Cu homeostasis proteins. **A.** Crystal structure of high-affinity Cu importer Ctr1 obtained from *Salmo salar*. Individual subunits highlighted in purple, cyan, and blue (PDB: 6M97). Metal-binding residues shown in ball- and stick representation in yellow. **B.** Crystal structure of human ATOX1 dimer bound to Zn^2+^ ion (PDB: 7ZC3). Individual monomers are shown in green or blue. Metal-binding cysteine residues are shown in a ball-and-stick representation in orange. Zn^2+^ ion is shown in white. **C.** Solution NMR structure of human metallothionein 2 bound to cadmium (Cd) ions (PBD: 1MHU). Metal-binding cysteine residues are highlighted in orange and Cd ions are depicted in gray.

Dietary absorption of Cu occurs through the apical side of intestinal epithelial cells mediated by the copper transporter 1 (CTR1), encoded by the *Slc31a1* gene (Fig. 3A). CTR1 expression is modulated by Cu availability, where limiting conditions results in the induction of the coding gene *Slc30a1* while Cu-replete conditions repress it [36–40]. From the gastrointestinal tract, Cu is transported to the liver and then secreted into the bloodstream bound to soluble chaperones [41–44] such as ceruloplasmin and albumin [10, 43, 45]. At the cell surface, these chaperones release Cu^2+^ to the metalloreductase, STEAP2, that converts the ion to its monovalent form (Cu^+^) for uptake by CTR1 [41]. Within target tissues and organs, distribution of Cu is mediated by intracellular Cu-binding chaperones and ligands, which coordinate delivery of Cu to specific organelles for utilization [46, 47]. The intracellular Cu-trafficking network includes high-affinity ligands, such as glutathione (GSH), and Cu chaperones and cuproenzymes such as antioxidant cytosolic copper chaperone 1 (ATOX1) (Figs 1 and 3B), cytochrome *c* oxidase copper chaperone 17 (Cox17), the Cu^+^ chaperone for superoxide dismutase, and metallothioneins (MTs) (Fig. 3C) [47–50].

ATOX1 is a key chaperone that delivers cytosolic Cu to soluble cuproproteins and proteins in the trans-Golgi network (TGN) and is essential for Cu excretion via ATP7A and ATP7B [50–54]. ATOX1 is structurally similar to the cytosolic metal-binding domains (MBD) of Cu^+^-ATPases (Fig. 3B) [55]. Working alongside ATOX1, the reduced form of GSH is an intracellular Cu ligand, chelating internalized metals to mitigate toxicity and aid in Cu uptake and MT binding [56–59]. GSH regulates ATOX1 by reducing glutathione disulfide (GSSG) and oxidizing the Cu^+^-binding site, influencing its redox state and metal affinity [58]. Produced in the cytosol, GSH is shuttled to various cellular compartments [60, 61], with mitochondrial GSH (mGSH) transported into the matrix via carriers like the 2-oxoglutarate carrier and the dicarboxylate carrier [62–68]. Mitochondrial ROS are converted to hydrogen peroxide (H_2_O_2_) by manganese superoxide dismutase [69, 70], and mGSH cycling, involving GSH peroxidases Gpxs and the nicotinamide adenine dinucleotide phosphate (NADPH)-dependent GSSG reductase (GR), plays a key role in H_2_O_2_ metabolism and membrane protection [71–76]. Lipid peroxide detoxification also involves GSH, Gpx4, and GR, protecting mitochondria from oxidative damage [63, 77–79].

**Figure 4.**
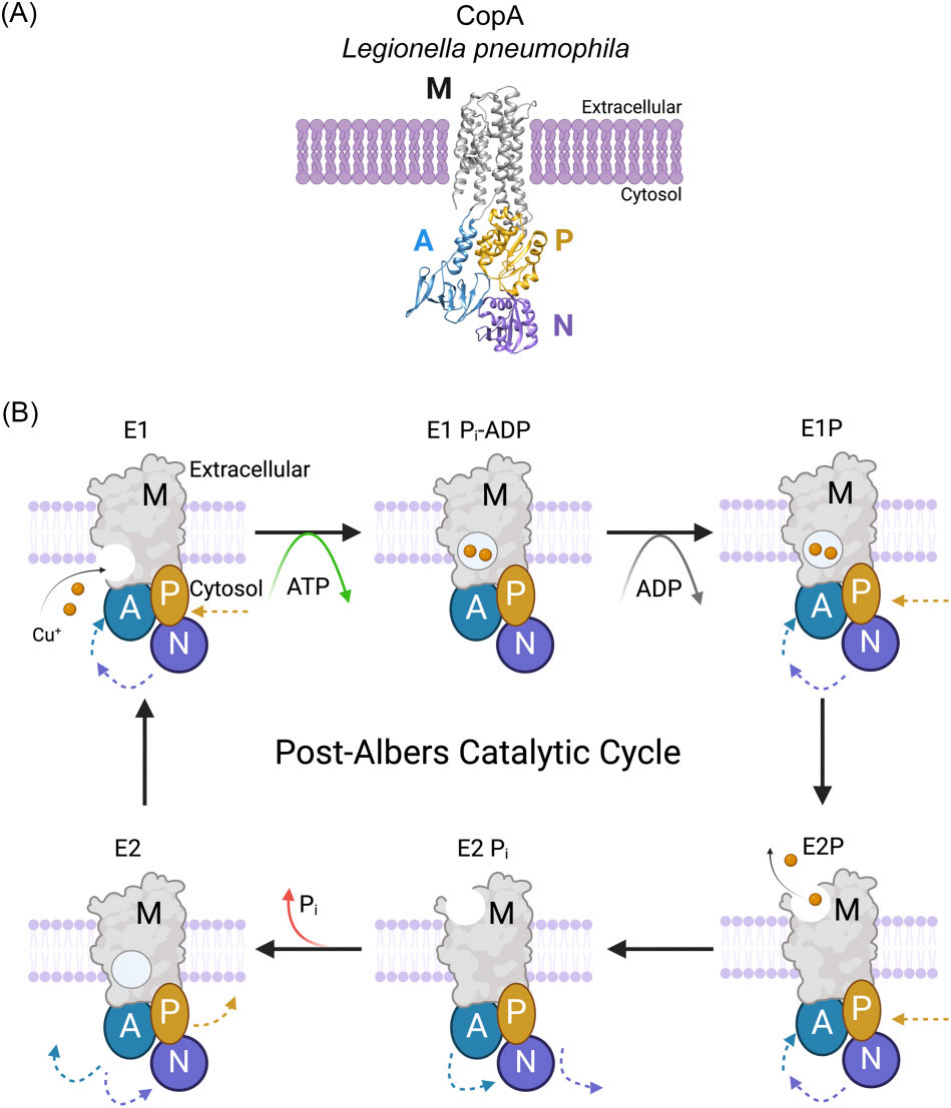
Structure and catalytic cycle of P-type Cu^+^ ATPases. **A.** Crystal structure of *Legionella pneumophila* CopA, the bacterial homolog of human Cu^+^-ATPases (PDB: 3RFU). Transmembrane domain (M) is depicted in gray. The catalytic phosphorylation domain (P), nucleotide-binding domain (N), and actuator domain (A) are depicted in gold, purple, and blue, respectively. **B.** Schematic representation of the Post–Albers E1–E2 catalytic cycle of Cu^+^ P-type ATPases. The ATPase binds Cu^+^ at high affinity during the E1 state. Conformational shifts of the intracellular domains occur following ATP hydrolysis and phosphorylation, which occludes the M domain in the E1P_i_-ADP state. Disassociation of ADP results in the E1P state, from which the ATPase transitions to the E2P state to release Cu^+^ to the extracellular side of the membrane. Inorganic phosphate is released to transition into the E2 state. Additional conformation change reopens the Cu^+^ entrance point on the cytosolic side to return the E1 state. Figure made with BioRender.

Cu excretion and mobilization via secretory vesicles and metalation of cuproenzymes is facilitated by homologous P_1B1_-type Cu^+^-ATPases ATP7A and ATP7B (Fig. 4A) [80–84]. These exporters couple Adenosine Triphosphate (ATP) hydrolysis to ion movement to control cytosolic Cu levels [10, 85–90] and protein metalation in tissues and subcellular locations based on Cu requirements and control system-wide Cu distribution [85, 91–94]. Structurally, both isoforms have eight transmembrane helices (MA, MB, and M1–M6) that contain several Cu^+^-coordinating residues or metal-binding sites (Fig. 4A) [95–98]. Eukaryotic Cu^+^-ATPases possess a highly conserved ‘CXXC’ N-terminal MBD, which independently folds from the rest of the protein and serves a structural role to facilitate and regulate ion uptake [91, 99, 100]. Cu^+^ transport by ATP7A and ATP7B follows the classic Post–Albers catalytic cycle proposed for P_2_-ATPases (Fig. 4B) [101–103].

Cu is also essential for mitochondrial energy production, acting as a prosthetic group of the cytochrome *c* oxidase (COX) complex. Mitochondrial Cu is mobilized by several cuproproteins including Cox17 and Cox11, synthesis of cytochrome *c* oxidase 1 and 2 (Sco1 and Sco2) and the phosphate and Cu^+^-transporter Slc25A3 (PiC2) to be delivered to COX (Fig. 1). COX is an essential enzyme in the electron transport chain, responsible for catalyzing the reduction of oxygen to water, enabling ATP production through oxidative phosphorylation. COX contains two Fe centers (heme *a* and heme *a_3_*) and two Cu centers (Cu_A_ and Cu_B_), which sequentially catalyze the reduction of oxygen (O_2_) to water (H_2_O) to terminate the respiratory chain [104, 105]. To metalate COX, the small chaperone Cox17 delivers Cu into the mitochondria where Cox11, Sco1, Sco2, and PiC2 will contribute to the maturation of the COX complex [106–111]. Delivery of Cu to the mitochondria is independent of COX, and it was proposed that the majority of mitochondrial Cu is in the matrix [112, 113]. Mobilization of Cu into the mitochondrial matrix was also shown to occur bound to a non-proteinaceous fluorescent ligand, termed CuL [110, 113, 114]. CuL levels were found to correlate with cellular Cu levels and COX assembly [110, 115]. CuL, suggested to be coordinated by citrate and oxaloacetate, migrates with a mass of ∼13 000 Da, hinting at larger ligands. Fluorescence anisotropy experiments showed that the CuL complex is transported by SLC25A3 and MRS3 transporters [115–117]. Although the molecular identity of CuL and the mechanism for exiting the matrix and delivering Cu to apo-COX17 in the intermembrane space remain unclear, its biophysical properties suggest it contributes to buffering cytosolic Cu and facilitating Cu uptake into mitochondria [110, 113]. PiC2 is capable of transporting Cu from both, the CuL purified from the mitochondrial matrix and ionic Cu from liposomes and the *Lactococcus lactis* system [116]. However, it remains unclear whether CuL is transported as an intact complex or whether Cu is released from the ligand during transport [116–118]. A recent study, using liquid chromatography with inductively coupled plasma mass spectrometry (ICP-MS), found no CuL-like species in cytosol or mitochondrial extracts in yeast [119]. Instead, the metallothionein CUP1 was the dominant Cu species in mitochondria from healthy cells, localizing to the intermembrane space [119]. The analyses explored cytosolic and mitochondrial Cu species from yeast cells cultured with varying Cu concentrations and detected over two dozen Cu species including both Cu proteins and nonproteinaceous complexes [119]. Competition assays revealed unexpected interactions between Cu-containing species, and experiments with *Cox17*-depleted cells showed no accumulation of potential trafficking precursors [119]. These experiments reveal stable Cu proteins and coordination complexes in the cytosol and distinguished those from the intermembrane space from those in the matrix/inner membrane [119].

## Cu-binding proteins as emerging transcriptional regulators of mammalian cell development

**Figure 5.**
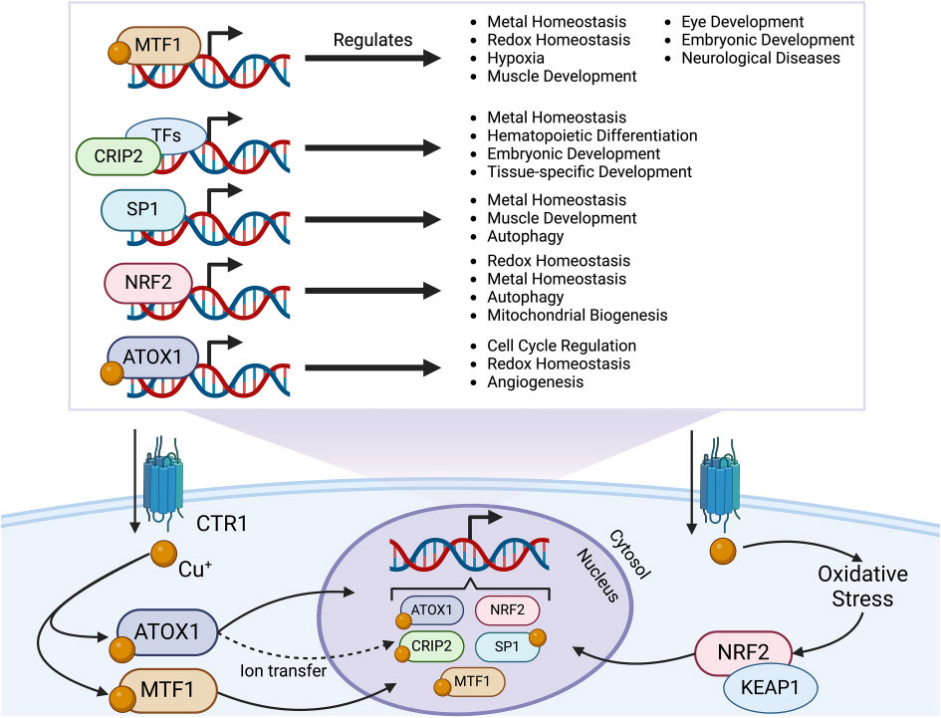
Summary of Cu-binding transcriptional regulators and their function. Cu-TFs and transcriptional regulators mediate a variety of biological functions in response to Cu. Classically, Cu-binding transcriptional proteins are involved in the maintenance of metal homeostasis, redox homeostasis, and hypoxic response. Additional roles for Cu-binding transcriptional proteins have been described in development. MTF1 is the main regulator of cellular metal homeostasis but is also implicated in the development of various tissues. SP1 is also a key Cu sensor in mammals and plays a role in Cu-dependent muscle development alongside of MTF1. ATOX1 is a widely studied Cu^+^ chaperone; however, recent studies have demonstrated Cu-dependent transcriptional activity for ATOX1 on targets involved in cell cycle regulation. NRF2 is a key regulator of cellular redox conditions and is responsive to Cu-induced oxidative stress. Finally, Crip2 is an emerging molecule that facilitates expression of genes associated to metal homeostasis and muscle development. Figure made with BioRender.

Limited information is available on Cu-binding proteins as transcription factors (Cu-TFs) and Cu-dependent transcriptional regulators; however, recent efforts have aimed to describe their function in this biological context (Fig. 5). These factors bind Cu predominately through histidine and cysteine motifs, similar to Cu chaperones and transporters. Cu binding might be a prerequisite for the regulatory function of these factors, allowing them to function as Cu sensors as well. Most of the research on Cu-TFs has been focused on their response to metal and redox dysregulation; however, emerging evidence showed novel roles for these proteins in normal cell development and as potential therapeutic agents for neurological diseases and injuries.

## ATOX1 functions as a Cu^+^ chaperone and Cu-dependent transcription factor

ATOX1 is a well-studied Cu chaperone proposed to act as a transcriptional regulator [120]. It is essential for the development of fibroblasts, vascular smooth muscle, endothelial, and neuronal cells [35, 51, 120–129]. In murine models, *Atox1* deletion results in perinatal lethality or developmental deficiencies, including immobility, hypothermia, and pigmentation loss [130]. Importantly, intravenous Cu supplementation in pregnant Atox1^+/−^ mice revealed that the chaperone is required for Cu transfer from the placenta to Atox1^−^^/^^−^ pups [130].

Beyond its chaperone role, Atox1 regulates processes like inflammation, cancer cell migration, and the cell cycle via transcriptional activation [121, 123, 127, 131]. Cu-dependent Atox1 homodimerization enables its transcriptional role at the *cyclin-D1* promoter, regulating the G1/S transition [132–137]. Cu-induced cell proliferation and *cyclin-D1* mRNA expression are diminished in Atox1^−/−^ cells but restored by re-expressing the wild-type human ATOX1 [131]. Conversely, Cu supplementation increased *cyclin-D1* promoter activity in wild-type but not Atox1^−/−^ cells, with electrophoretic mobility shift assay (EMSA) and chromatin immunoprecipitation (ChIP) studies showing Cu-stimulated Atox1 binding to the promoter [131, 132]. However, fluorescence microscopy and *in vitro* binding studies suggest Atox1 may act through indirect protein interactions [138]. Atox1 also regulates *Sod3* expression through Cu-dependent transcription [121]. EMSA and ChIP experiments revealed that Atox1 binds a GAAGA sequence in the *Sod3* promoter, which is similar to its recognition site at the *cyclin-D1* promoter [121]. This dual role in cell cycle and redox regulation presents a novel Cu-dependent mechanism [121, 131, 132]. In vascular development and inflammation, Atox1 facilitates Cu delivery to Atp7A, which activates lysyl oxidase (LOX), an enzyme essential for angiogenesis [123, 139–141]. Additionally, Atox1 has been proposed to function as a Cu-TF for NADPH oxidase (*p47phox*), which promotes ROS production and inflammatory neovascularization [123, 142, 143]. Reporter assays linked Atox1 to increased promoter activity of *p47phox*, promoting monocyte adhesion and inflammatory response [123]. In summary, Atox1 has potential roles in Cu-dependent transcriptional regulation of genes involved in the cell cycle, redox homeostasis, and angiogenesis [121, 123, 124, 131, 132]. Given its involvement in cancer and atherosclerosis, understanding its transcriptional regulation mechanisms is crucial for therapeutic targeting [127, 144–147].

## Specificity protein 1 is an oscillating Cu sensor

Specificity protein 1 (Sp1) is a widely expressed transcription factor that regulates numerous genes involved in cell growth and function across various tissues [148–155]. Knockout (KO) of *Sp1* in murine embryos is lethal, with its expression profile varying across different cell types and developmental stages [150, 153, 155]. Mechanistically, Sp1 recruits the transcription factor II D (TFIID) precomplex to gene promoters, facilitating the recruitment of RNA polymerase [156, 157]. Additionally, Sp1 can repress gene expression by interacting with the epigenetic modifier histone deacetylase 1 (HDAC1) at promoters such as p21, thereby regulating cell cycle progression in proliferating cells [158, 159]. Sp1 is particularly enriched in hematopoietic and myeloid cells, highlighting its contributions to cell cycle regulation across diverse cell types [155, 160–162].

**Figure 6.**
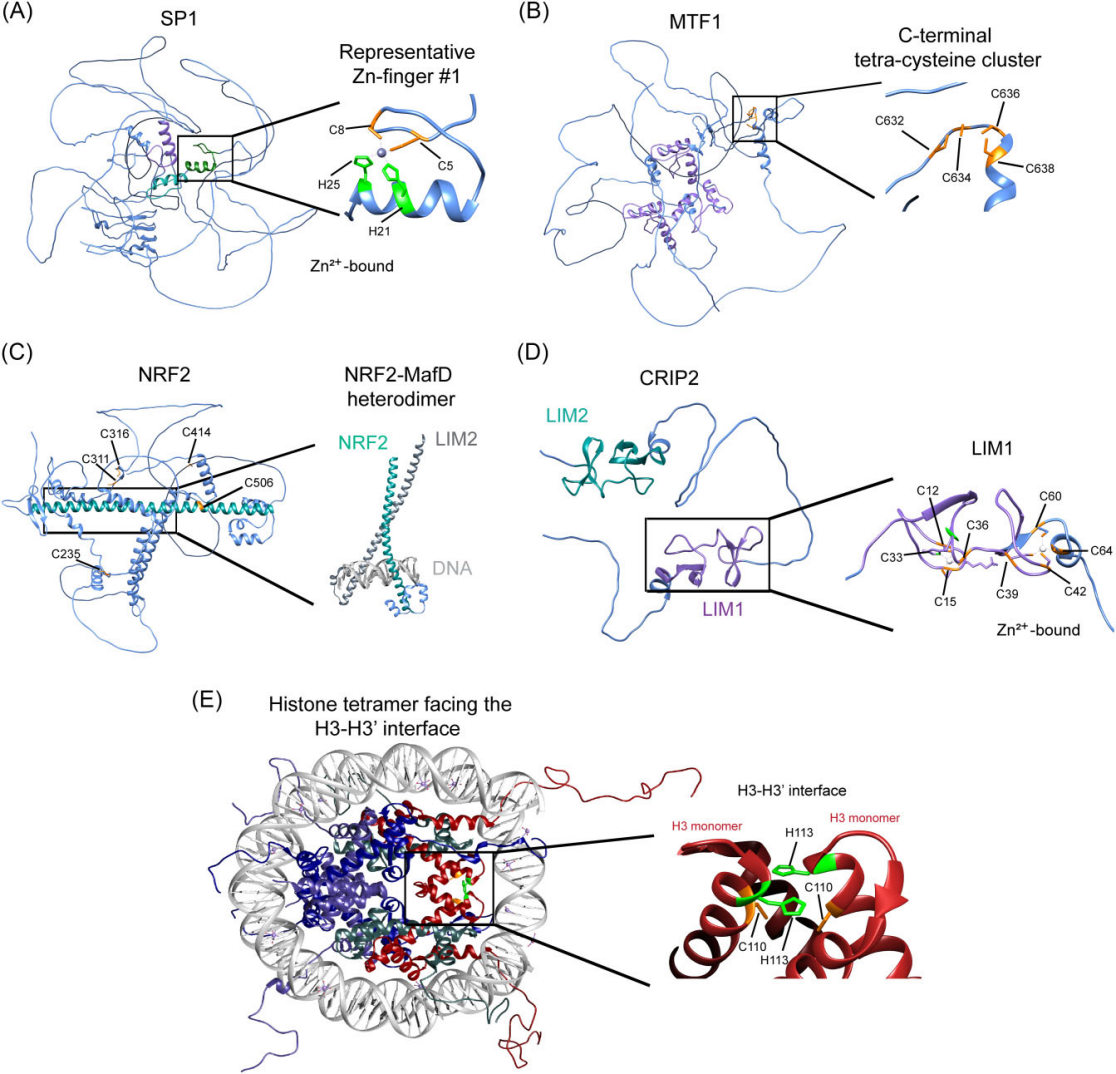
Protein structures of copper-binding transcription factors. **A.** SP1: Left panel shows a predictive AlphaFold model of human SP1 with its zinc-finger (ZF) motifs highlighted in green, cyan, and purple. The NMR structure of a representative ZF bound to Zn (PDB: 1SP1) is also shown. Key metal-binding residues are represented as ball and stick, with histidines in green and cysteines in orange. Right panel zooms in on a ZF of SP1 coordinating one Zn²⁺ ion. **B.** MTF1: Left panel presents a predictive AlphaFold model of human MTF1 with ZF motifs highlighted in purple. Right panel zooms in on the Cu⁺-coordinating C-terminal tetracysteine cluster. **C.** NRF2: Left panel shows a predictive AlphaFold model of human NRF2 alongside the crystal structure of the NRF2-MafD heterodimer bound to DNA (PDB: 7 × 5F), with reactive cysteines in orange. Right panel zooms in on the interaction regions of NRF2 (green), MafD (dark gray), and DNA (light gray). **D.** CRIP2: Left panel shows a predictive AlphaFold model of CRIP2, while the right panel presents the NMR structure of the human CRIP2 LIM1 domain bound to Zn (PDB: 2CU8). Metal-binding histidines and cysteines are highlighted in green and orange, respectively. **E.** Nucleosome: Left panel displays the X-ray structure of a *Xenopus laevis* nucleosome tetramer facing the H3–H3′ interface (PDB: 1KX5), with H2A in navy, H2B in purple, H3 in red, and H4 in gray. Right panel zooms in on the H3–H3′ interface, showing Cu coordination by cysteine and histidine residues (green and orange, respectively, in ball and stick).

Within the cellular Cu network, Sp1 functions as a Cu-sensor and transcriptional regulator of Cu transporters *Slc30a1* (encoding CTR1) and *ATP7A* in cultured human and rat cells [40, 163–166]. Sp1 contains a Zn-finger (ZF) domain with two serine/threonine-rich and two glutamine-rich regions at the N-terminus, essential for its activation and multimerization. An additional ZF with high affinity for Cu is located at the C-terminus, enabling Sp1 to act as a Cu-folding protein (CFP) that modulates DNA-binding activity (Fig. 6A) [157, 164, 166–168]. In its CFP form, Sp1 does not bind the *Slc31a1* promoter, suggesting that Cu binding alters its regulatory function, but increased Sp1 levels correlate with higher Slc31a1 expression. Cu depletion induces Sp1 expression, while Cu abundance suppresses it, through Cu-dependent binding of Sp1 to its own promoter, thereby mediating *Slc31a1* expression in response to Cu levels [40, 165].

Sp1 and ATP7A engage in a Cu-dependent feedback loop, regulating each other's expression in response to cellular Cu levels [163, 164, 169]. In zebrafish embryos, silencing *Atp7a* results in decreased Sp1 expression, indicating this regulatory interplay [169]. In rat intestinal epithelial (IEC-6) cells, Sp1 cooperates with hypoxia-inducible factor alpha (Hif2α) to regulate *Atp7A* expression, particularly under hypoxic and Fe-deficient conditions. Hif2α, a key regulator of cellular respiration and metabolism activated by oxygen depletion, works with Sp1 to induce *Atp7A*, as well as Fe transport genes *Dmt1* and *Dcytb* [163, 164, 170–174]. Although the exact mechanism remains unclear, it is plausible that Cu replaces Zn in Sp1, affecting its interaction with Hif2α and the *Atp7a* promoter, as both Sp1 and *Atp7a* are repressed under Cu-replete conditions [165, 175]. This suggests a complex regulatory mechanism to modulate Cu and Fe homeostasis and hypoxia, where Fe deficiency triggers Hif2α target expression, including *Atp7a*, linking Cu and Fe metabolism through redox processes [176]. In summary, Sp1 is an essential transcription factor that serves as a Cu sensor, regulating Cu and Fe homeostasis through the transcriptional control of key transporters and interactions with other regulatory proteins like Hif2α. Its roles extend to cell cycle regulation, redox homeostasis, and angiogenesis, making Sp1 integral to the metal homeostasis network and a potential therapeutic target for related pathologies such as cancer and atherosclerosis.

## Metal regulatory transcription factor 1 mediates cuprostasis and mammalian cell development

The cellular response to metal and redox alterations is orchestrated by metal regulatory transcription factor 1 (MTF1) through the recognition of metal-responsive elements (MREs) in promoter regions of target genes [177–180]. MTF1 regulates the expression of genes related to metal and redox homeostasis, cell proliferation, and tissue development in response to a variety of metals, including Zn, Cd, and Cu [177–179, 181–189]. MREs are *cis-*acting DNA elements found in metal-regulated genes comprised of an imperfectly conserved 12-base pair motif—with a highly conserved 7-base pair central motif (5′-TGCRCNC) followed by a less conserved 5-base pair GC-rich motif (5′-GGCCC) [180, 190]. The DNA-binding domain of MTF1 is made of six CCHH-type ZFs that are thought to mediate the Zn-sensing function (Fig. 6B) [178, 191–193]. The individual ZFs control different functions of MTF1, with ZF#1 involved in Zn-dependent DNA-binding with Sp1, and ZFs#5 and #6 determining MRE-binding specificity [180, 194, 195]. Nonconventional linker sequences between the ZF domains contribute to nuclear localization, DNA binding, and transcriptional activity [196, 197]. Finally, the C-terminal domain of MTF1 is important for Cu-dependent dimerization and transcriptional activation [189, 198–200]. Metal binding leads to the nuclear translocation of MTF1 to promote transcription of target regulatory genes, such as MTs and transporters [189, 201–203]. Cd binding does not activate Mtf1 but induces Mt expression, which was proposed to be an indirect consequence of increased Zn concentration due to Cd displacement [56, 180].

MTF1 is highly conserved across species. Studies from mammalian cells and *Drosophila melanogaster* (dMtf1) evidenced key similarities and differences in how Mtf1 and MREs operate between these organisms. For example, in both mammals and *Drosophila*, Zn is a primary activator of Mtf1 that induces nuclear translocation, where it binds to MREs to induce transcription of MTs and other metal-responsive genes to regulate homeostasis and detoxification [204]. However, the main differences noted between the mammalian and the *Drosophila* Mtf1 are in response to Cu. Early *in vitro* experiments with mammalian Mtf1 showed a poor activation by Cu under normal conditions, suggesting a more Zn-specific response. However, dMtf1 presents a carboxy-terminal tetranuclear cysteine cluster [205], which is conserved in mammalian cells, that has been shown to bind Cu and to be essential for transcriptional activation [206, 207]. *In vitro* assays showed differences on whether Zn or Cu ions directly induce the binding of Mtf1 to MREs in the promoter regions of target genes [208, 209]. The data led to a model where the structural changes in the ZFs of MTF1 drive a specific mechanism by which the factor binds to DNA in response to increasing Zn concentrations. In contrast, *in vitro* analyses showed a reduced response of Mtf1 to Cu stimulation, which was proposed to indirectly regulate Mtf1 by altering MT-bound Zn pools rather than binding directly to the transcription factor itself. However, *in vitro* studies may fail to account for the cellular components, signaling, and the complex dynamic metal homeostasis seen in cells or *in vivo*. The tight regulation of metal availability is mediated by transporters and chaperones, which may influence the activation of Mtf1 differently from what is observed *in vitro.* Furthermore, in metal-binding studies, especially in the context of Mtf1 and MREs, *in vitro* experiments often reveal valuable insights about the direct interactions between metal ions and transcription factors, yet translating these findings to *in vivo* conditions can be challenging. In this sense, ChIP-seq analyses performed by our group showed that, in primary myoblasts stimulated with non-toxic concentrations of Cu, Mtf1 presented an increased binding to different genes involved in both, metal homeostasis and skeletal muscle differentiation, supporting a relevant role for Cu and Mtf1 in muscle development [189]. Moreover, through site-directed mutagenesis, our group showed that ablation of the C-terminal Cu^+^-binding site on Mtf1 impaired the DNA-binding and transcriptional capabilities of Mtf1 and of the myogenic factor 1, in the context of skeletal muscle differentiation [189]. Mtf1 also regulates mitochondrial cuprostasis in muscle cells by transcriptionally activating *Slc25a3*, which transports inorganic phosphate and Cu into the mitochondrial matrix for COX activity [116–118]. Disruption of *Slc25a3* leads to muscle pathologies due to impaired ATP synthesis, including lactic acidosis and hypertrophic cardiomyopathy [210, 211]. ChIP experiments in primary myoblasts revealed Mtf1 binding to the *Slc25a3* promoter, containing MREs, with Cu supplementation enhancing this interaction, suggesting Cu-dependent gene regulation [184, 189]. Loss of *Slc25a3* impaired cell proliferation and differentiation by disrupting mitochondrial function and lowering mitochondrial cuproprotein expression, highlighting the role of Mtf1 in mitochondrial cuprostasis and COX function during muscle development [184]. While Mtf1 may directly bind Cu to activate transcription, its primary action involves Zn binding, with factors like MTs regulating cuprostasis.

Cumulative evidence demonstrates the relevance of Mtf1 for embryonic development in mice, fruit flies, and zebrafish, as well as for tissue-specific development in the liver and skeletal muscle cells [179, 189, 212, 213]. Mice embryos lacking *Mtf1* fail to express *Mt1* and *Mt2* and have reduced expression of the heavy chain subunit of γ-glutamylcysteine synthetase, a subunit of the enzyme that produces GSH [178, 179]. In murine models, heterozygous *Mtf1*^+/−^ animals presented a normal appearance, but when intercrossed, most of the homozygous *Mtf1*^−/−^ pups were not viable [179]. *Mtf1*^−/−^ 13.5-day-old embryos showed no morphological differences from the heterozygous or wild-type littermates. However, histological analysis of KO embryos showed severe liver damage due to congested sinusoids and disassociation of epithelia accompanied by decreased cytokeratin expression in liver cells [178]. By 14.5 days of embryonic development, the expression of cytokeratin was almost completely abolished in surviving animals, which presented liver necrosis and generalized edema alongside extensive DNA fragmentation [179]. Hepatocytes isolated from 12.5-day-old *Mtf1*^−/−^ embryos exhibited signs of necrosis upon culture and died a couple of days after extraction [213]. These cells lacked classical markers of liver differentiation, such as hepatocyte nuclear factor 4, α-fetoprotein, and carbamoyl phosphate synthetase [213]. Reintroduction of the *Mtf1* gene into KO zygotes restored embryonic viability, providing direct evidence of its contributions to development [213]. Contrasting results between adult and embryonic *Mtf1*^−/−^ specimens showed that deletion of this gene is not fatal in mature individuals. Silencing of *Mtf1* in adult mice resulted in normal liver function but elevated sensitivity to Cd and Zn, as cells failed to express *Mt1, Mt2*, and the Zn transporter *Znt1* [213]. The data support a model where, in adult individuals, Mtf1 mainly regulates targets involved in metal homeostasis, while during embryonic growth, this Cu-TF may regulate additional developmental pathways.

Consistent with this idea, studies with dMtf1 showed a broader metal-sensing ability, including a more significant role in Cu homeostasis, possibly because Cu is more crucial for certain developmental stages in *Drosophila*. dMtf1 has a dual role in cuprostasis by regulating *Mt* expression in response to Cu overload and Ctr1B when Cu is limited [188]. *dMtf1* KO impaired transcription of *Ctr1B*, one of three Ctr1 isoforms essential for Cu uptake and rapid growth during larval development [188, 214, 215]. This deletion resulted in elevated sensitivity to Cu depletion and thus, altered larval development. While *dMtf1* KO does not block the development of the flies, as is the case in *Mtf1* null mice [179, 216], the adult flies were sensitive to Cu variations [178–180, 203, 214], a phenotype that was rescued by introduction of the human protein [217].

In both *Drosophila* and mammalian cells, MTF1 regulates the expression of the Cu^+^-ATPases DmATP7 (the sole isoform of ATP7A/B in flies) and ATP7A and ATP7B, respectively [218]. In human hepatocellular carcinoma (HepG2 and HLE) cells, EMSA experiments demonstrated that MTF1 binds to an MRE in the *ATP7B* suggesting a role in cuprostasis by controlling Cu export [183]. This was further supported by a case study of a WD patient with a 379-bp homozygous variant (chr12.52, 586, 149T>C) in the *ATP7B* promoter, which disrupts MTF1 binding and results in the WD phenotype [182]. Luciferase assays in HepG2 cells showed reduced *ATP7B* reporter expression in cells with the WD variant compared to healthy controls, with MTF1 overexpression partially restoring *ATP7B* expression, though less effectively in the WD variant [182]. ChIP experiments confirmed that MTF1 binds to the affected *ATP7B* promoter region, and disruption of this binding site likely underlies the WD phenotype [182]. This binding site is crucial for MTF1-mediated ATP7B function, highlighting the role of MTF1 in Cu excretion and its potential as a biomarker and therapeutic target for WD.

Thus, it is possible that mammalian cells may rely also on other Cu-regulatory proteins like ATOX1 and CTR1, but the emerging roles of MTF1 as a Cu-dependent regulator suggests the existence of additional mechanisms related to development. However, the precise mechanism of the potential Cu-dependent activation of mammalian Mtf1 in cell differentiation remains less clear compared to Zn and is yet to be characterized. It is plausible that, depending on the cellular context, Mtf1 responds directly to Cu or through intermediary molecules like MTs, ATOX1, and CRIP2 (see later). Thus, there is still much to explore about how Cu modulates Mtf1 activity directly, not only in terms of cuprostasis, but also in terms of mammalian cell development and differentiation.

## Contributions of MTF1 to the development of the eye and the nervous system

Additional experiments regarding animal development using zebrafish embryos as a model showed that expression of a dominant negative *Mtf1* version reduced transcription of genes involved in eye development, brain and central nervous system (CNS) development, and nuclear receptors [219]. Interestingly, the expression of the eye protein α-crystallin was repressed in these animals, and several MRE sequences were identified at its promoter region [219]. α-crystallin is a major lens protein in the eye, which has structural and chaperone roles that protect against aggregates that damage the eye [220–224]. Importantly, the promoter of *α-crystallin* is responsive to Zn, Cd, and Cu in human and zebrafish cells, suggesting a mechanism by which metals and MTF1 may regulate eye development and homeostasis, as well [219, 225].

Mtf1 is proposed to regulate transcription of genes involved in the development and remediation of neurological conditions. Examples of these are the major prion protein (PrP) and β-synuclein (β-syn) [13, 14]. PrP is the causative protein of transmissible spongiform encephalopathies (also known as prion diseases), which include fatal neurogenerative pathologies, such as Creutzfeldt–Jakob disease, kuru, and Gerstmann–Sträussler–Scheinker syndrome [226–232]. The propagation of prion disease depends on the conformational interconversion of PrP between two isoforms: host-encoded cellular prion protein (PrP^C^) and the pathogenic (PrP^SC^) [233]. Both isoforms maintain the same primary sequence and are encoded by the *PRNP* gene; expression of PrP^C^ is a prerequisite for the manifestation of the pathological phenotype [230, 234, 235]. Structurally, PrP^C^ is mainly constituted by α-helices, while PrP^SC^ is principally β-sheets, the latter results in detergent and protease-resistant neurotoxic aggregates [236, 237]. PrP^C^ is a glycoprotein linked to the plasma membrane by a glycosylphosphatidylinositol anchor, highly expressed in neuronal and glial cells and in portions of nonneuronal tissue and may be linked to cuprostasis [227, 236, 238–241]. PrP^C^ has two Cu-binding domains, where Cu binding at the primary domain induces endocytosis of the receptor [230, 242–246]. *In vitro* experiments have shown that Cu^2+^ is reduced to Cu^+^ at the N-terminal region of PrP^C^, and it is observed that PrP^C^ null cerebellar cells have decreased levels of Cu [230, 247, 248], suggesting that PrP^C^ may play a role to maintain cuprostasis in neuronal cells [247, 248].

Studies in cultured rat hippocampal and cortical neurons revealed that the Prnp promoter contains three MREs, with PrP^C^ expression induced by Cu and Cd, but not Zn. Cu chelation with bathocuproine sulphonate reduced PrP^C^ expression, suggesting a direct link between Cu and Prnp regulation [14, 249]. However, detergent extracts from hippocampal neurons showed no protease-resistant PrP^SC^, indicating that Cu may not be involved in PrP^C^/PrP^SC^ interconversion [249]. The mechanism by which Cu transcriptionally regulates *Prnp* activation is thought to occur through promoter interactions with Mtf1 and Sp1. Experiments with immortalized human fibroblasts from an MD patient (MNK cells) explored the role of MTF1 and SP1 in regulating PRNP expression [14, 250–252]. MNK cells lacking *ATP7A* (MNK^(Del)^) had elevated Cu levels, while MNK cells expressing *ATP7A* (MNK^+/+^) had lower Cu content [14, 15, 250, 251]. Both MNK^(Del)^ and control cells expressed PrP^C^, but MNK^+/+^ cells did not [14]. Transfecting MNK^(Del)^ cells with MTF1 and/or SP1 increased *PRNP* transcript and PrP^C^ protein levels, further enhanced by Cu supplementation, while MNK^+/+^ cells showed no PrP^C^ expression [14]. These findings suggest MTF1 and SP1 transcriptionally regulate *PRNP* via a Cu-activated model, linking PrP to Cu homeostasis and highlighting MTF1 as a potential therapeutic target for prion diseases.

β-Synuclein is another target of MTF1 implicated in neurodegenerative diseases. Synucleins are a family of three intrinsically disordered proteins: α-synuclein (α-syn), β-synuclein (β-syn), and γ-synuclein (γ-syn) expressed in a variety of neurons and other tissues [253–256]. Synucleins have been extensively investigated due to their role in the development of neurodegenerative disorders, termed synucleinopathies [257]. α-Syn is the most widely studied member of the synuclein family as it is the causative protein in Parkinson's disease, dementia with Lewy bodies, and multiple system atrophy [258, 259]. These neuropathies are characterized by misfolding of α-syn resulting in the accumulation of large neurotoxic aggregates [260, 261]. More recently, research in neurodegenerative disease has paid more attention to β-syn, as the expression of β-syn in blood and cerebrospinal fluid appears to be a prognostic marker for synaptic damage independent of synucleopathy [254, 262, 263]. While the physiology and pathophysiology of α-syn have been a subject of significant research efforts, the physiological role of β-syn and γ-syn is not well characterized yet. It has been observed, however, that α-syn and β-syn colocalize to presynaptic nerve terminals where they regulate vesicle trafficking, inhibit dopamine synthesis, and induce extracellular dopamine scavenging in dopaminergic neurons [264–271].

MTF1 binds to an MRE identified in Exon 1 of the *β-syn* gene, approximately at 1.1–0.6 kb downstream of the transcription starting site [13]. Experiments in the SH-SY5Y human neuroblastoma cell line overexpressing promoter fragments containing the β-syn MRE and MTF1 showed increased promoter activity compared to controls [13]. Site-directed mutagenesis experiments where the MRE site was perturbed and in cells cultured under Cu-deplete conditions showed that the activity of the *β-syn* promoter was abolished regardless of MTF1 overexpression [13]. EMSA and transcriptional analyses demonstrated that overexpression of MTF1 increased binding to the MRE sequence in the *β-syn* promoter, which resulted in increased β-syn expression but no other synucleins [13]. These results suggest that activation of the *β-syn* promoter occurs, at least in part, through a Cu-dependent mechanism of MRE recognition by MTF1.

The regulation of β-syn by MTF1 suggests a neuroprotective role for MTF1, as β-syn counteracts α-syn aggregation by activating neuroprotective pathways, reducing α-syn accumulation and competing for shared substrates [272–283]. Evidence suggests that expression of α-syn and β-syn is maintained by a tight transcriptional regulatory mechanism, wherein an increase in β-syn expression reduces α-syn levels [284]. While metal-binding activity has been observed for all synucleins, Cu^+^ binding only promotes α-syn aggregation [13, 285–292]. In this regard, β-syn was proposed as a protective Cu-chelating agent to prevent Cu-induced α-syn aggregation [262]. Thus, it is plausible that β-syn has a positive role in neuronal cuprostasis, which might be mediated by Cu-dependent activation by MTF1 [13]. In the case of synucleinopathies, the metal toxicity and oxidative stress response facilitated by MTF1 may be disrupted, leading to failure in β-syn neuroprotection and subsequent oligomerization of α-syn protein [13, 272, 274, 276, 293].

MTF1 has also been identified as a neuroprotective regulator of the Na^+^/Ca^2+^ exchanger 1 (NCX1), which is expressed in all CNS cell types and maintains ion homeostasis by balancing Na^+^ and Ca^2+^ concentrations [294–296]. NCX1 operates as a secondary transporter, coupling the inward Na^+^ gradient with Ca^2+^ export [297]. During ischemic events, such as strokes, ATP depletion impairs Na^+^/K^+^ ATPase function, causing NCX1 to reverse and pump Ca^2+^ into cells [298, 299]. In animal models of ischemic injury, *NCX1* silencing worsens brain damage, highlighting it as a potential target for neuroprotective therapies [300]. Studies on stroke remediation leverage the neuroprotective function of NCX1 by modulating its associated transcriptional regulators [294, 298, 301–303]. Computational and EMSA analyses of the *NCX1* promoter identified MRE sequences and binding sites for Hif-1α and Sp1 [294, 301, 302]. In a murine stroke model using transient middle cerebral artery occlusion (tMCAO), NCX1 protein levels decreased by ∼50%, and Mtf1 expression was significantly reduced [294]. This suggests a connection between Mtf1 and NCX1 during ischemic insult, which could be targeted to mitigate stroke damage. Remote limb ischemic postconditioning (RLIP), involving temporary ischemia applied to the femoral artery, has shown promise in reducing brain damage from ischemia [304–306]. Murine models of traumatic brain injury and stroke demonstrated that RLIP triggers neuroprotective pathways and preserves cognitive functions and motor coordination [294, 306–310]. RLIP restored Mtf1 and NCX1 expression after tMCAO, and *Mtf1* knockdown (KD) in femoral artery occlusion-treated tMCAO mice diminished the neuroprotective effects of RLIP, highlighting the importance Mtf1 in this process [294].

Current evidence emphasizes the involvement of Mtf1 in metal homeostasis, redox changes, hypoxia response, and its roles in embryonic development, myoblast differentiation, and the regulation of synucleins and α-crystalline. The broad biological significance of Mtf1 makes it a promising target for understanding and treating various physiological and pathological conditions.

## NRF2 is a key regulator of oxidative stress caused by Cu

The complex regulation of oxidative stress and cuprostasis has been experimentally linked due to the redox potential of Cu [311]. NRF2, a member of the Cap‘n'Collar (CNC) subfamily of basic-region leucine-zipper transcription factors (Fig. 6C), plays a crucial role in oxidative stress protection and signaling, and drug metabolism [312–314]. The CNC family also includes NRF1, NRF3, Bach1, and Bach2, all of which interact with antioxidant response elements (AREs) in target gene promoters [315–326]. AREs, similar to MREs targeted by MTF1, consist of a 16-base pair consensus sequence (5′-TMAnnRTGAYnnnGCR-3′; M = A or C, Y = C or T, W = A or T) [120, 312, 318–321, 327–334]. NRF2 acts as a redox sensor through conserved cysteine residues (C119, C235, and C506), and mutations in these residues impair its ability to bind AREs [335]. In the absence of oxidative stress, NRF2 is bound and inhibited by Keap1, a redox-sensing protein, which maintains NRF2 in the cytosol for ubiquitination and degradation by Cul3 E3 ligase. Keap1 has cysteine residues (C257, C273, C288, and C297) that sense oxidation states; thus, modifications by ROS lead to conformational changes, reducing NRF2-Keap1 interactions [336–339]. Upon release, NRF2 translocates to the nucleus and forms a heterodimer with small musculoaponeurotic fibrosarcoma proteins (Mafs), binding to ARE sequences to activate the ‘Phase 2 detoxification response’ [340, 341]. This response induces the expression of enzymes like glutathione S-transferases (GST) and NAD(P)H-reductase, and increases reduced GSH levels, thereby protecting against oxidative stress [340–342].

NRF2 plays a crucial role in cuprostasis by regulating *Mt* expression through its interaction with the ARE sequence. In the *Mt1* promoter, an ARE at −101 bp overlaps with a upstream factor 1 (USF1)-binding site [343–345]. Studies showed that H_2_O_2_ induces *MT1* transcription in human liver endothelial cells (Hepa) via both ARE and MRE, reflecting responses to metal and oxidative stress through independent mechanisms [346–349]. Deleting the ARE reduces *MT* responses to H_2_O_2_ and Cd but not Zn [350]. In CD-1 rats, long-term Cu exposure upregulated NRF2-Keap1 signaling in kidneys, causing oxidative damage and kidney malfunction, especially at higher Cu concentrations, where decreased antioxidant capacity worsened damage [351]. Thus, it has been proposed that the mechanism by which Cu activates NRF2 is through redox activity rather than metal binding, in a process that involves redox cycling agents such as hydroquinones [352, 353]. For example, tert-butylhydroquinone (tBHQ), a known NRF2 activator, is oxidized by Cu^2+^ to form quinones that can activate NRF2 by modifying reactive thiol groups in Keap1 [354, 355]. In AREc32 cells, Cu enhances NRF2 activation in the presence of tBHQ, with dose-dependent increases in luciferase activity. Cu chelators, tetraethylenepentamine (TEPA) and deferoxamine, inhibit this activation, emphasizing the role of Cu in NRF2 activation through redox cycling [352, 356].

NRF1, another CNC transcription factor, regulates *MT1* along with NRF2 [323, 357]. NRF1 and NRF2 have overlapping functions in antioxidant defense but differ in their cellular mechanisms and localization. NRF1 is mainly involved in lipid metabolism and liver function and is anchored to the endoplasmic reticulum [358]. In *Nrf1*-null mice, NRF2 and its downstream targets, including *GST1* and *NAD(P)H dehydrogenase quinone 1*, are upregulated, indicating NRF2 compensates for NRF1 loss [323]. Furthermore, in *Nrf1* and *Nrf2* double KO mice, canonical NRF2 targets are completely silenced, highlighting the dependency on NRF2 for antioxidant responses in the absence of NRF1 [323]. NRF1 and NRF2 bind to the *MT1* ARE with similar affinity; however, NRF1 has stronger activity in *MT1* promoter assays compared to NRF2 [323]. Studies using fibroblasts from Nrf1-null (Nrf1^−/−^) and Nrf2-null (Nrf2^−/−^) mice showed increased sensitivity to Cu for both, with Nrf2^−/−^ cells being more sensitive at higher Cu concentrations [357]. Luciferase reporter assays revealed different responses of *Mt1* to Cu in these KO cells. Nrf1^−/−^ cells exhibited reduced basal *Mt1* expression but increased activity with Cu supplementation, while Nrf2^−/−^ cells showed no change in Mt1 expression [357]. This discrepancy may be due to putative NRF1-binding sites in the *Mt1* promoter not present in the reporter construct [357]. Transcriptional analyses showed increased *Mt1* mRNA levels in Nrf1^−/−^ cells post-Cu exposure, whereas Nrf2^−/−^ cells also showed elevated *Mt1* mRNA levels, contrary to reporter assay results. These findings suggest NRF1 and NRF2 regulate *Mt1* expression under Cu and oxidative stress through distinct mechanisms [357].

Additionally, NRF2 links oxidative stress with lipid metabolism and autophagy, by interacting with sequestosome-1 (p62), Beclin1, and microtubule-associated proteins 1A/1B [359–364]. An ARE located at the promoter of *p62*, an autophagy receptor, is regulated by Nrf2, which also influences peroxisome proliferator-activated receptor-γ (PPARγ) upon Cu-induced redox stress [365–369]. Studies with yellow catfish fed a high Cu diet showed increased liver triglycerides, elevated Nrf2 levels, and decreased Keap1, alongside upregulated lipogenic enzymes and downregulated lipolytic genes [364]. Cu enhanced the nuclear translocation of NRF2 and PPARγ expression, with Nrf2 binding directly to the PPARγ promoter [364]. This interaction is crucial for lipid metabolism and adipocyte differentiation, suggesting Nrf2 as a potential therapeutic target for oxidative stress-related conditions like nonalcoholic fatty liver disease, diabetes, and obesity.

Beyond these roles, NRF2 is involved in drug detoxification and various pathologies [313, 370–375]. It plays a protective role in detoxification processes, such as those triggered by acetaminophen overdose, and is crucial in countering lung diseases caused by cigarette smoke [370, 371, 376–378]. NRF2 also has a complex role in cancer, providing both resistance and promoting pathogenesis, depending on the tumor context [372, 379–388]. Thus, NRF2 is a vital regulator in the Cu network, influencing oxidative stress responses and potentially serving as a target for therapeutic intervention in multiple diseases associated with oxidative stress, such as nonalcoholic fatty liver disease, diabetes, obesity, and cancer [363, 389–392].

## CRIP2 is a Cu-binding transcriptional regulator

Recent studies from our laboratory evidenced a novel transcriptional regulatory role for the Cu-binding protein cysteine-rich intestinal protein 2 (CRIP2, Fig. 6D) [50, 53]. CRIP2 is a member of the CRIP family of evolutionary conserved LIM domain proteins (named after the first three genes the domain was discovered in: Lin-11, Isl-1, and Mec-3) [53]. CRIP1 contains a single LIM domain, while CRIP2 and CRIP3 contain 2 LIM domains (Fig. 6D) [53]. The LIM domains of CRIP2 and CRIP3 are separated by a largely disordered region of amino acids and adopt a dual ZF motif (Fig. 6D) [53]. These isoforms have an N-terminal (Zn1) and a C-terminal (Zn2) LIM domain, with CxxC…HxxC and CxxC…CxxC motifs, respectively [53]. Human and murine CRIP2 and CRIP3 conserve the Zn1 and Zn2 motifs, with the addition of conserved histidine residues near the Zn2 site [53]. CRIP2 binds both Zn and Cu with high affinity at a 4:1 stoichiometric ratio [50, 53]; however, the purified recombinant protein can also bind Cu and Zn simultaneously in a 2:2:1 stoichiometry [53]. The binding of Cu to CRIP2 seems to be mediated by cysteine residues. ICP-MS experiments on a cysteine-to-serine mutant of CRIP2 (CRIP2^CBS^) presented lower Cu binding [50]. Cyclic voltammetry experiments of the wild-type protein showed limited redox activity, which may be important for protein–protein interactions, or additional cellular processes where it might be involved [53].

The physiological roles of CRIP2 are not well described; however, reports have linked CRIP2 autophagy and glycolytic inhibition in the H1299 cancer cell line, alongside skeletal muscle and cardiovascular development [50, 53, 393]. CRISPR/Cas9-mediated deletion of *Crip2* in murine primary myoblasts reveals that while Crip2 is essential for differentiation, it is not required for proliferation [53]. *Crip2* KO cells exhibit increased apoptosis and impaired tissue organization, which can be reversed by reintroducing the human wild-type gene [53]. Although Cu treatment partially rescues cell viability in *Crip2* KO cells, it does not correct the differentiation defect, and elevated Cu levels are found in vesicles of these cells [53]. Since Crip2 lacks a DNA-binding domain, it is proposed that its interaction with gene promoters is through indirect chromatin associations [53]. Proteomic analysis identified Crip2 interacting with Atox1, suggesting a role in Cu transfer and nuclear translocation [50]. Cu affects Crip2 differently depending on the cell type; it causes degradation in H1299 cells but increases Crip2 expression in differentiating myoblasts. Cu chelation with TEPA reduces Crip2 levels and affects differentiation [50, 53].

In myoblasts, Crip2 shows significant enrichment at Sreb2 motifs in the absence of Cu, and at RUNX1 and ZBTB12 motifs in the presence of Cu, impacting genes related to myogenesis and Cu-dependent mitochondrial function [53]. RNA-seq analyses indicate *Crip2* KO affects genes related to mRNA processing, chromatin organization, and muscle development [53]. Crip2 also appears to act as a metal sensor, binding to promoters of Zn transporters and *MTs*. Its role in autophagy is suggested by increased LC3II levels and decreased p62 in *Crip2* KO H1299 cells, indicating a role in suppressing autophagy [50]. Cu binding is crucial for this function, as autophagy is restored by reintroducing wild-type Crip2 but not a Cu-binding mutant [50]. Overall, the diverse roles of Crip2 across cell types and conditions, particularly its involvement in transcriptional regulation and metal homeostasis, highlight the need for further research into its regulatory mechanisms and physiological functions.

## The H3–H4 histone tetramer is a Cu-binding enzyme

Histones, traditionally recognized for their role in chromatin organization, are now proposed to possess additional enzymatic functions. The H3–H4 tetramer, a fundamental histone complex, exhibits metalloreductase activity by catalyzing the reduction of Cu²⁺ to Cu⁺ in *Saccharomyces cerevisiae* and *Xenopus laevis* [16, 394, 395]. This discovery reveals a novel function of histones beyond their DNA-packing role and opens new research avenues into their enzymatic capabilities. Cu binding occurs at the H3–H3′ interface of the tetramer, facilitated by conserved cysteine (C110) and histidine (H113) residues (Fig. 6E) [395]. ultraviolet (UV) absorption spectroscopy and isothermal titration calorimetry have demonstrated strong Cu binding, with C110 being particularly critical for this interaction [395]. Specifically, UV absorbance changes due to Cu²⁺ binding are disrupted by mutation of C110, indicating its crucial role [395]. The reductase activity of the H3–H4 tetramer requires an electron donor such as tris(2-carboxyethyl)phosphine (TCEP), nicotinamide-adenine dinucleotide (NADH), or NADPH but is not affected by the presence of oxygen [395]. This activity is specific to Cu ions, as demonstrated by the faster reduction rate of Cu²⁺ in the presence of these donors compared to natural conditions [395]. In yeast models, mutations affecting Cu-binding and reductase activity have significant implications [395]. For instance, the H3H113A mutation is lethal, abolishes reductase activity, and reduces the retention of the H3–H4 tetramer on Cu²⁺ affinity columns. Mutations such as H3H113N or H3H113Y, while non-lethal, disrupt Cu²⁺ binding and lead to altered Cu⁺ levels, resulting in slow-growing strains [395]. These mutations also affect the expression of Cu-binding activator 1 (Mac1) mRNA, suggesting that altered Cu levels might be responsible for changes in gene expression [395]. Furthermore, experiments in yeast lacking the high-affinity Zn transporter (Zrt1) resulted in reduced enzymatic activity of the yeast H3–H4 tetramer [394]. The H3–H4 tetramer impacts transcription of yeast metallothionine (Cup1), wherein Zrt1 deletion decreases activity of Cup1 reporter genes. Cu1 expression is mediated by the Cup2 TF, which is sensitive to the oxidation state of Cu, an indication of Cu^2+^ accumulation due to lower H3–H4 reductase activity [394].

Interestingly, the Cu²⁺-reductase activity of the H3–H4 tetramer has been implicated in the pathophysiology of FRDA, a progressive neurodegenerative disorder caused by mutations in the frataxin gene (*FXN*) [16]. Fe–S clusters, which are critical for cellular function, are affected by the balance between their production and degradation [16]. Given that Cu⁺ and Fe–S clusters compete for the mitochondrial biosynthesis site, the H3–H4 reductase activity could influence Fe–S cluster biosynthesis [16]. In yeast models of FRDA, where the frataxin homolog Yfh1 is deleted, the H3H113N mutation partially restores growth and function, suggesting that it might mitigate the effects of disrupted Fe–S cluster biosynthesis [16]. Additionally, H3H113N mutation helps to reduce toxic Cu⁺ levels, supporting its role in alleviating FRDA-related cellular stress [16].

Overall, the Cu²⁺-reductase activity of the H3–H4 tetramer highlights a novel enzymatic function of histones, contributing to Cu homeostasis and influencing cellular processes related to metal ion regulation. This function is dependent on electron donors and is enhanced by Zn [394, 395]. Cysteine and histidine residues mediate Cu binding and redox capabilities, and mutations affecting these residues impair the enzymatic function [395]. The involvement of the H3–H4 tetramer in diseases like FRDA [16] evidences the importance of understanding its catalytic roles and how they impact metal-mediated transcription and gene regulation. Furthermore, the Cu-binding capability of DNA-binding proteins like histones opens frontiers for new mechanisms of Cu-mediated transcription and regulation of gene expression.

## Conclusions

Cu is a vital co-factor for cellular and tissue function, with its levels tightly controlled by a complex network of Cu-binding proteins, transporters, and Cu-TFs. Traditionally, research has concentrated on mechanisms of cuprostasis and the consequences of Cu dysregulation. The focus has often been on Cu-TFs and their role in regulating cuprostasis through transcriptional mechanisms. However, emerging evidence suggests that Cu and Cu-binding proteins play previously unrecognized roles in cell and tissue development. The progress in this field highlights the need for deeper exploration into the involvement of Cu in transcriptional regulation. Cu is implicated in various human diseases, including cancer and neurological disorders. Expanding our knowledge of Cu-binding proteins in the context the transcription regulation of these pathologies could open new diagnostic and therapeutic possibilities, particularly for rare conditions such as MD and WD. Future research should employ innovative techniques to further uncover how Cu influences transcriptional regulation and contributes to disease mechanisms.

## Data Availability

This review is based on previously published studies and does not include any original data. All data referenced in this manuscript are available from the cited articles, which are publicly accessible or can be obtained from the respective authors or repositories.
